# 非手术干预的肺腺癌患者发生脑膜转移的危险因素分析

**DOI:** 10.3779/j.issn.1009-3419.2025.101.06

**Published:** 2025-04-20

**Authors:** Yi YUE, Yuqing REN, Jianlong LIN, Chunya LU, Nan JIANG, Yanping SU, Jing LI, Yibo WANG, Sihui WANG, Junkai FU, Mengrui KONG, Guojun ZHANG

**Affiliations:** 450000 郑州，郑州大学第一附属医院; The First Affiliated Hospital of Zhengzhou University, Zhengzhou 450000, China

**Keywords:** 肺肿瘤, 脑膜转移, 危险因素, 肝转移, EGFR L858R突变, Lung neoplasms, Meningeal metastasis, Risk factors, Liver metastases, EGFR L858R mutation

## Abstract

**背景与目的:**

脑膜转移（meningeal metastasis, MM）是一种由肿瘤细胞从原发部位转移至软脑膜、硬脑膜、蛛网膜、蛛网膜下腔及其他脑脊液间隙的恶性肿瘤转移形式。肺癌是MM最常见的恶性肿瘤类型之一。MM不仅表明肺癌已进展至晚期，而且由于脑膜的受累，将会引起一系列严重的临床症状。本研究旨在探究非手术干预的肺腺癌（lung adenocarcinoma, LUAD）患者发生MM的危险因素，以尽可能识别出具有发生MM高危风险的非手术干预的LUAD患者。

**方法:**

本研究回顾性分析2020年1月至2024年7月于郑州大学第一附属医院诊断为LUAD的患者的临床资料，使用多重插补的方法处理缺失数据，通过LASSO、单因素及多因素Logistic回归方法进行危险因素分析。

**结果:**

研究共纳入170例LUAD患者，分为两组，其中出现MM 87例，未出现MM 83例，单因素及多因素Logistic回归结果显示确诊LUAD时的年龄（P=0.004）、携带表皮生长因子受体（epidermal growth factor receptor, EGFR）基因21外显子L858R突变（P=0.008）、基线时合并肝转移（P=0.004）是非手术干预的LUAD患者发生MM的独立危险因素，而基线时具有较高的球蛋白（globulin, GLB）水平（P=0.039）以及携带间变性淋巴瘤激酶（anaplastic lymphoma kinase, ALK）基因突变（P=0.040）的患者罹患MM的风险较小。

**结论:**

确诊LUAD时的年龄、EGFR L858R和ALK基因的突变状态、基线合并肝转移和基线GLB水平与晚期LUAD患者罹患MM的风险密切相关，临床上对于确诊LUAD时年龄较小、携带EGFR L858R基因突变、基线时合并肝转移的非手术干预的LUAD应尽早实施MM的三级预防措施，并定期监测患者的MM状态。

## Abstract (en)

**Background and objective:**

Meningeal metastasis (MM) is a form of malignant metastasis where tumor cells spread from the primary site to the pia mater, dura mater, arachnoid, subarachnoid space, and other cerebrospinal fluid compartments. Lung cancer is one of the most common malignant tumor types with MM. MM not only signifies that the lung cancer has progressed to an advanced stage but also leads to a range of severe clinical symptoms due to meningeal involvement. Currently, the risk factors associated with the development of MM are not fully elucidated. The aim of this study was to investigate the risk factors for MM in patients with lung adenocarcinoma (LUAD) who underwent non-surgical interventions, in order to identify LUAD patients at high risk for MM.

**Methods:**

This retrospective study analyzed the clinical data of patients diagnosed with LUAD at the First Affiliated Hospital of Zhengzhou University from January 2020 to July 2024. Missing data were imputed using multiple imputation methods, and risk factors were identified through LASSO, univariate, and multivariate Logistic regression analyses.

**Results:**

A total of 170 patients with LUAD were included in this study and divided into two groups: 87 patients with MM and 83 patients without MM. Univariate and multivariate Logistic regression analyses revealed that younger age at diagnosis (P=0.004), presence of the epidermal growth factor receptor (EGFR) L858R gene mutation (P=0.008), and concurrent liver metastasis at baseline (P=0.004) were independent risk factors for developing MM in LUAD patients who did not undergo surgical intervention. Conversely, higher baseline globulin levels (P=0.039) and the presence of the anaplastic lymphoma kinase (ALK) gene mutation (P=0.040) were associated with a reduced risk of MM development.

**Conclusion:**

Age at diagnosis, EGFR L858R mutation status, ALK gene mutation status, concurrent liver metastasis, globulin levels at baseline were significantly associated with the risk of developing MM in patients with LUAD patients who did not undergo surgical intervention. For patients diagnosed at a younger age, carrying the EGFR L858R mutation, or presenting with baseline liver metastasis, early implementation of tertiary prevention strategies for MM is crucial. Regular monitoring of MM status should be conducted in these high-risk groups.

脑膜转移（meningeal metastasis, MM）是一种由肿瘤细胞从原发部位转移至软脑膜、硬脑膜、蛛网膜、蛛网膜下腔及其他脑脊液间隙的恶性肿瘤转移形式。MM可以继发于脑转移、与脑转移同时发生，也可以先于脑转移发生^[1]^。MM不仅标志着癌症已进入晚期，而且由于脑膜的受累，会引起一系列严重的临床症状，如恶心、呕吐、吞咽困难、大小便失禁、癫痫发作和类似中风的表现，显著降低患者的生活质量，并危及生命。几乎所有类型的恶性肿瘤都可以出现MM。肺癌是全球发病率第一的恶性肿瘤，也是MM最常见的恶性肿瘤类型之一，其MM发病率为3.4%-10.8%^[1,2]^。近年来，肺癌的治疗进展已使某些类型的肺癌，特别是具有高基因突变频率和可治疗靶点选择的肺腺癌（lung adenocarcinoma, LUAD），逐渐转变为一种可长期管理的慢性疾病。此外，随着对MM关注度的提升以及检测技术的迅速发展和普及，MM的检出率逐年上升。尽管早期治疗能够显著延长MM患者的总生存期，但却常常忽视对高危患者的早期识别。早期识别出具有MM高危风险因素的患者并及时实施三级预防措施，不仅可以显著延长患者的总生存期，还能提高其生活质量。迄今为止，仅有少数研究探讨了肺癌患者发生MM的危险因素，这些研究主要集中在酪氨酸激酶抑制剂（tyrosine kinase inhibitors, TKIs）治疗后的肺癌患者中。此外，MM好发于LUAD患者。因此，本研究聚焦于非手术干预的LUAD患者这一特定群体，以确保对危险因素的分析能够与LUAD患者的临床特征和治疗反应保持高度一致，从而为个性化治疗提供更坚实的基础。本研究纳入170例LUAD患者的临床资料数据，通过关注LUAD患者发现基线时的年龄、表皮生长因子受体（epidermal growth factor receptor, EGFR）L858R和间变性淋巴瘤激酶（anaplastic lymphoma kinase, ALK）基因突变状态、肝转移和球蛋白（globulin, GLB）水平与非手术干预的LUAD患者罹患MM的风险密切相关，特别对于确诊LUAD时年龄较小、携带EGFR L858R基因突变、基线时合并肝转移的非手术干预的LUAD患者要采取更加积极的干预措施。

## 1 资料和方法

### 1.1 一般资料

回顾性分析符合纳排标准的2020年1月至2024年7月于郑州大学第一附属医院诊断为MM的LUAD患者以及同时期诊断为LUAD的非MM患者的临床资料，包括：基线时（初次抗肿瘤治疗前）的年龄、性别、实验室检查（血常规、肝功能、肾功能、凝血功能、电解质等）、影像学检查[胸部计算机断层扫描（computed tomography, CT）、颅脑增强磁共振成像（magnetic resonance imaging, MRI）、正电子发射断层显像/CT（positron emission tomography/CT, PET/CT）、彩色多普勒超声、放射性核素骨扫描等]、MM状态、基因突变类型以及出现MM前的抗肿瘤治疗方式等。所有患者的随访时间均超过18个月，或者在随访期间出现了MM。本研究的主要结局指标为颅脑增强MRI显示MM或脑脊液细胞学检查发现肿瘤细胞。本研究获得郑州大学第一附属医院伦理委员会批准（批准号：2023-key-0390-002），按照《赫尔辛基宣言》原则进行。鉴于其回顾性性质，不需要患者知情同意。

### 1.2 纳入与排除标准

纳入标准：（1）经病理细胞学或组织学确诊的LUAD；（2）年龄>18岁；（3）基线临床资料、实验室检查、影像学等资料完整。排除标准：（1）存在其他部位原发肿瘤；（2）存在其他病理类型的肺癌，如腺鳞癌；（3）基线临床资料、实验室检查、影像学等资料不完整；（4）年龄≤18岁；（5）既往行开颅手术；（6）初诊时即合并MM。MM的诊断标准参照欧洲神经肿瘤学会和欧洲内科肿瘤学会（European Association of Neuro-Oncology-European Society for Medical Oncology, EANO-ESMO）神经肿瘤学临床实践指南和美国临床肿瘤学会（American Society of Clinical Oncology, ASCO）指南所示^[3,4]^。这些指南规定，脑脊膜转移的诊断标准为脑脊膜MRI上脑脊神经、脑沟、小脑叶和脊髓上的线状或结节状沉积物等典型MRI表现，必要时结合神经影像学检查后脑脊液中肿瘤细胞的鉴定。所有的MRI报告及脑脊液病理结果均由郑州大学第一附属医院的两名副高级医师独立进行审核。

### 1.3 分析指标

本研究主要收集并分析患者首次确诊LUAD时的一般资料、实验室检查、影像学检查以及基因突变类型等指标与MM发生的关系，具体如下：（1）一般资料：性别、年龄、肿瘤分期以及MM前的治疗方案。（2）实验室检查：白细胞（white blood cell, WBC）、红细胞（red blood cell, RBC）、血红蛋白（hemoglobin, HB）、血小板（platelet, PLT）、中性粒细胞（neutrophils, NEU）、淋巴细胞（lymphocytes, LYM）、单核细胞（monocytes, MON）、红细胞压积（hematocrit, HCT）、平均红细胞体积（mean corpuscular volume, MCV）、平均红细胞血红蛋白含量（mean corpuscular hemoglobin, MCH）、平均红细胞血红蛋白浓度（mean corpuscular hemoglobin concentration, MCHC）、平均血小板体积（mean platelet volume, MPV）、血小板压积（platelet crit, PCT）、凝血酶原时间（prothrombin time, PT）、凝血酶原活动度（prothrombin activity, PTA）、国际标准化比率（international normalized ratio, INR）、活化部分凝血活酶时间（activated partial thromboplastin time, APTT）、纤维蛋白原（fibrinogen, FIB）、凝血酶时间（thrombin time, TT）、D-二聚体（D-dimer）、钾、钠、氯、钙、磷、葡萄糖（glucose, GLU）、尿素氮（blood urea nitrogen, BUN）、肌酐（creatinine, CR）、尿酸（uric acid, UA）、谷丙转氨酶（alanine aminotransferase, ALT）、谷草转氨酶（aspartate aminotransferase, AST）、γ-谷氨酰转肽酶（gamma-glutamyl transferase, GGT）、碱性磷酸酶（alkaline phosphatase, ALP）、总蛋白（total protein, TP）、白蛋白（albumin, ALB）、GLB、总胆红素（total bilirubin, TBIL）、直接胆红素（direct bilirubin, DBIL）、间接胆红素（indirect bilirubin, IBIL）、总胆固醇（cholesterol, Chol）、甘油三酯（triglycerides, TG）、低密度脂蛋白（low-density lipoprotein, LDL）、肾小球滤过率（glomerular filtration rate, GFR）。（3）影像学检查：肿瘤的位置、大小，肝脏、脑实质、颅骨、下肢骨、上肢骨、椎骨以及胸廓骨的转移情况以及是否合并肾上腺结节/增生、纵隔淋巴结肿大、颈部淋巴结肿大、癌性淋巴管炎、阻塞性肺炎等。（4）基因突变类型：EGFR 19外显子缺失（EGFR exon 19 deletion, EGFR 19Del）、EGFR T790M、EGFR L858R突变、EGFR 20外显子插入突变、鼠类肉瘤病毒癌基因（kirsten rat sarcoma viral oncogene, KRAS）基因突变、肿瘤蛋白53（tumor protein p53, TP53）基因突变、磷脂酰肌醇3激酶催化亚单位α（phosphatidylinositol-4,5-bisphosphate 3-kinase catalytic subunit alpha, PIK3CA）突变、ALK基因突变、视网膜母细胞瘤蛋白1（retinoblastoma protein 1, RB1）基因突变、鼠类肉瘤滤过性毒菌致癌同源体B（v-raf murine sarcoma viral oncogene homolog B, BRAF）基因突变、c-ros原癌基因（c-ros oncogene 1 receptor kinase, ROS1）突变、红细胞白血病病毒癌基因同源物（erythroblastic leukemia virus B, ERBB）基因突变、EGFR扩增、转染重排（rearranged during transfection, RET）原癌基因突变、间质-上皮细胞转化因子（mesenchymal-epithelial transition factor, MET）基因突变情况。

### 1.4 统计学分析

本研究共纳入170例LUAD患者。使用R编程语言，排除缺失值超过10%的变量。对于剩余的88个变量，使用“mice”函数进行多重插补，连续变量使用“pmm”方法进行插补，二分类变量使用“Logreg”方法进行插补，插补次数为5次。采用卡方检验、确切概率法和秩和检验评估组间差异。分类变量以百分比（%）表示，连续变量使用中位数（Q1, Q3）进行展示。采用“glmnet”程序包进行LASSO回归分析，将LASSO回归分析结果中惩罚系数不为0的变量纳入单因素及多因素Logistic回归进行分析。在所有在单因素分析中P<0.05的变量纳入多因素分析，统计显著性水平设定为α=0.05。

## 2 结果

### 2.1 患者临床特征

在2143例LUAD患者中，我们排除了1973例不符合纳入标准的患者（图1）。通过腰椎穿刺术抽取脑脊液行液基薄层细胞学检查找到癌细胞和/或MM MRI组合序列确定是否存在MM。共有170例患者纳入本研究，我们收集了年龄、性别、基线时肿瘤的分布、大小、转移部位、基因突变类型、实验室检查指标以及MM前的治疗方案等变量，剔除缺失值比例超过10%的变量，最终有88个变量被纳入分析。表1和图2A呈现了变量的缺失情况。如表2所示，多重插补处理前后，所有变量的P值均大于0.05，表明数据在插补前后具有可比性。表3展示了MM组患者与非MM组患者的基线资料。基线资料表显示两组在肺部病灶的分布位置（P=0.009）、基线时的MCHC（P=0.036）、TT（P=0.019）、PTA（P=0.046）、ALP（P=0.044）、DBIL（P=0.025）水平、基线时是否合并肾上腺结节或增粗（P=0.047）、下肢骨转移（P=0.004）、椎骨转移（P=0.016）、癌性淋巴管炎（P=0.007）、阻塞性肺炎（P=0.009）、肝转移（P<0.001），EGFR L858R突变（P=0.026）、TP53突变（P=0.041）、ALK突变（P=0.002）以及MM前是否应用免疫检查点抑制剂治疗（P=0.032）方面具有显著差异。

**图1:**
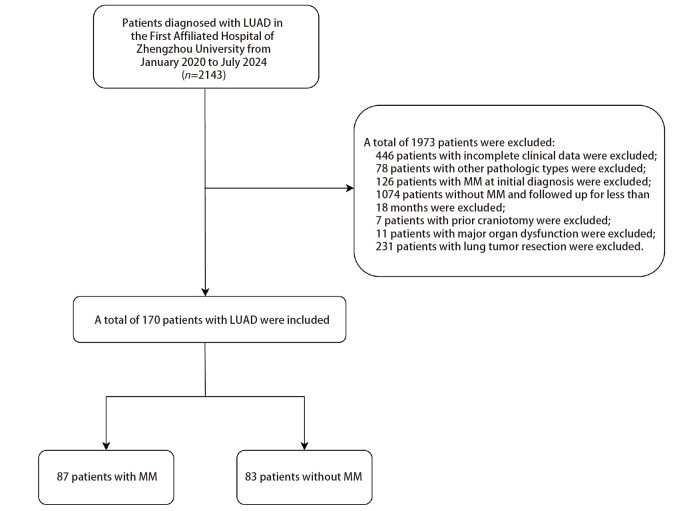
病例筛选流程图

**表1:** 170例LUAD患者重要变量的缺失情况

Variables	Missing cases (n)	Missing ratio (%)
Tumor location	4	2.35
Right lung cancer	4	2.35
Tumor length	9	5.29
Brain metastases	1	0.59
Adrenal hyperplasia/nodules	2	1.18
Upper limb bone metastasis	2	1.18
Lower limb bone metastasis	2	1.18
Pelvic bone metastasis	1	0.59
Thoracic bone metastasis	1	0.59
Cervical lymph node metastasis	3	1.76
Liver metastasis	1	0.59

**图2:**
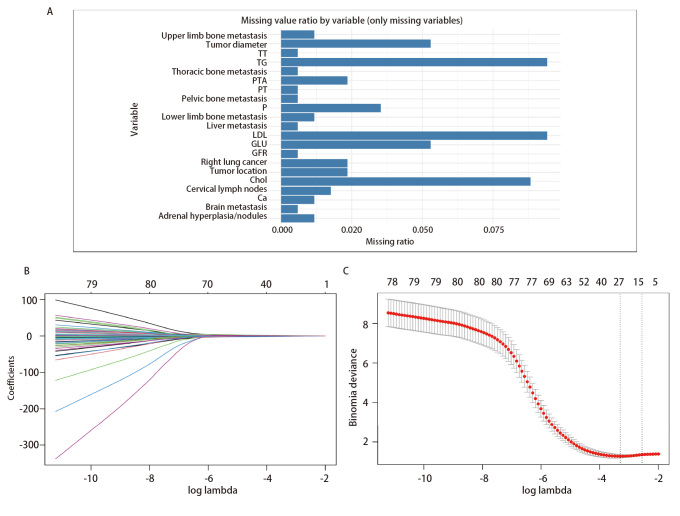
缺失变量及LASSO回归图。A：多重插补前缺失变量比例柱状图；B：LASSO回归系数收敛路径图；C：10折交叉验证图。

**表2:** 170例LUAD患者多重插补前后均衡性检验

Variables	Pro-imputation*	Post-imputation	Statistic	P
Tumor diameter, M (Q₁, Q₃) (mm)	35.00 (24.00, 51.00)	34.50 (24.00, 51.00)	Z=-0.07	0.940
Tumor location, n (%)			χ²=0.00	0.996
Central type	85 (50.00)	87 (51.18)		
Peripheral type	81 (47.65)	83 (48.82)		
Right lung cancer, n (%)			χ²=0.02	0.899
No	90 (52.94)	91 (53.53)		
Yes	76 (44.71)	79 (46.47)		
Brain metastases, n (%)			χ²=0.01	0.929
No	124 (72.94)	124 (72.94)		
Yes	45 (26.47)	46 (27.06)		
Adrenal hyperplasia/nodules, n (%)			χ²=0.02	0.900
No	148 (87.06)	149 (87.65)		
Yes	20 (11.76)	21 (12.35)		
Upper limb bone metastasis, n (%)			χ²=0.00	0.945
No	129 (75.88)	130 (76.47)		
Yes	39 (22.94)	40 (23.53)		
Lower limb bone metastasis, n (%)			χ²=0.05	0.831
No	134 (78.82)	134 (78.82)		
Yes	34 (20.00)	36 (21.18)		
Pelvic bone metastasis, n (%)			χ²=0.00	0.967
No	106 (62.35)	107 (62.94)		
Yes	63 (37.06)	63 (37.06)		
Thoracic bone metastasis, n (%)			χ²=0.01	0.940
No	112 (65.88)	112 (65.88)		
Yes	57 (33.53)	58 (34.12)		
Cervical lymph node metastasis, n (%)			χ²=0.00	0.945
No	91 (53.53)	92 (54.12)		
Yes	76 (44.71)	78 (45.88)		
Liver metastasis, n (%)			χ²=0.00	0.985
No	150 (88.24)	151 (88.82)		
Yes	19 (11.18)	19 (11.18)		

*Due to missing values in certain variables, the sample size before interpolation was fewer than 170.

**表3:** 170例LUAD患者基线资料表

Variables	Total cohort (n=170)	Non-MM group (n=83)	MM group (n=87)	Statistic	P
General information					
Age, M (Q₁, Q₃)	57.00 (52.00, 63.75)	58.00 (54.00, 65.00)	57.00 (50.00, 62.00)	Z=-1.86	0.063
Gender, n (%)				χ²=0.18	0.669
Female	95 (55.88)	45 (54.22)	50 (57.47)		
Male	75 (44.12)	38 (45.78)	37 (42.53)		
Clinical stage, n (%)				χ²=0.63	0.959
Stage I-II	16 (9.41)	8 (9.64)	8 (9.20)		
Stage III	22 (12.94)	9 (10.84)	13 (14.94)		
Stage IV	132 (77.65)	66 (79.52)	66 (75.86)		
Imaging data					
Tumor location, n (%)				χ²=6.77	0.009
Central type	87 (51.18)	34 (40.96)	53 (60.92)		
Peripheral type	83 (48.82)	49 (59.04)	34 (39.08)		
Right lung cancer, n (%)				χ²=0.23	0.629
No	91 (53.53)	46 (55.42)	45 (51.72)		
Yes	79 (46.47)	37 (44.58)	42 (48.28)		
Multiple nodules in both lungs, n (%)				χ²=0.79	0.373
No	109 (64.12)	56 (67.47)	53 (60.92)		
Yes	61 (35.88)	27 (32.53)	34 (39.08)		
Brain metastassis, n(%)				χ²=0.11	0.736
No	125 (73.53)	62 (74.70)	63 (72.41)		
Yes	45 (26.47)	21 (25.30)	24 (27.59)		
Adrenal hyperplasia/nodules, n (%)				χ²=3.93	0.047
No	149 (87.65)	77 (92.77)	72 (82.76)		
Yes	21 (12.35)	6 (7.23)	15 (17.24)		
Upper limb bone metastasis, n (%)				χ²=1.63	0.202
No	130 (76.47)	67 (80.72)	63 (72.41)		
Yes	40 (23.53)	16 (19.28)	24 (27.59)		
Lower limb bone metastasis, n (%)				χ²=8.10	0.004
No	134 (78.82)	73 (87.95)	61 (70.11)		
Yes	36 (21.18)	10 (12.05)	26 (29.89)		
Vertebral metastasis, n (%)				χ²=5.77	0.016
No	101 (59.41)	57 (68.67)	44 (50.57)		
Yes	69 (40.59)	26 (31.33)	43 (49.43)		
Cancerous lymphangitis, n (%)				χ²=7.28	0.007
No	156 (91.76)	81 (97.59)	75 (86.21)		
Yes	14 (8.24)	2 (2.41)	12 (13.79)		
Obstructive pneumonia, n (%)				χ²=6.87	0.009
No	126 (74.12)	69 (83.13)	57 (65.52)		
Yes	44 (25.88)	14 (16.87)	30 (34.48)		
Liver metastases, n (%)				χ²=12.56	<0.001
No	151 (88.82)	81 (97.59)	70 (80.46)		
Yes	19 (11.18)	2 (2.41)	17 (19.54)		
Variables	Total cohort (n=170)	Non-MM group (n=83)	MM group (n=87)	Statistic	P
Treatment before MM					
Local treatment, n (%)				χ²=0.00	1.000
No	161 (94.71)	79 (95.18)	82 (94.25)		
Yes	9 (5.29)	4 (4.82)	5 (5.75)		
Radiotherapy, n (%)				χ²=0.00	0.957
No	165 (97.06)	80 (96.39)	85 (97.70)		
Yes	5 (2.94)	3 (3.61)	2 (2.30)		
Chemotherapy, n (%)				χ²=0.19	0.666
No	33 (19.41)	15 (18.07)	18 (20.69)		
Yes	137 (80.59)	68 (81.93)	69 (79.31)		
Targeted therapy, n (%)				χ²=0.12	0.729
No	35 (20.59)	18 (21.69)	17 (19.54)		
Yes	135 (79.41)	65 (78.31)	70 (80.46)		
High BBB permeability TKIs drug therapy, n (%)				χ²=1.81	0.178
No	103 (60.59)	46 (55.42)	57 (65.52)		
Yes	67 (39.41)	37 (44.58)	30 (34.48)		
Immunotherapy, n (%)				χ²=4.60	0.032
No	129 (75.88)	57 (68.67)	72 (82.76)		
Yes	41 (24.12)	26 (31.33)	15 (17.24)		
Anti-vascular therapy, n (%)				χ²=0.92	0.338
No	76 (44.71)	34 (40.96)	42 (48.28)		
Yes	94 (55.29)	49 (59.04)	45 (51.72)		
Craniocerebral radiotherapy, n (%)				χ²=1.03	0.309
No	159 (93.53)	76 (91.57)	83 (95.40)		
Yes	11 (6.47)	7 (8.43)	4 (4.60)		
Genetic mutation status					
EGFR 19Del, n (%)				χ²=0.01	0.918
No	112 (65.88)	55 (66.27)	57 (65.52)		
Yes	58 (34.12)	28 (33.73)	30 (34.48)		
EGFR L858R, n (%)				χ²=4.93	0.026
No	113 (66.47)	62 (74.70)	51 (58.62)		
Yes	57 (33.53)	21 (25.30)	36 (41.38)		
ALK, n (%)				χ²=9.48	0.002
No	158 (92.94)	72 (86.75)	86 (98.85)		
Yes	12 (7.06)	11 (13.25)	1 (1.15)		
TP53, n (%)				χ²=4.16	0.041
No	139 (81.76)	73 (87.95)	66 (75.86)		
Yes	31 (18.24)	10 (12.05)	21 (24.14)		
RB1, n (%)				-	0.497
No	168 (98.82)	83 (100.00)	85 (97.70)		
Yes	2 (1.18)	0 (0.00)	2 (2.30)		
BRAF, n (%)				χ²=0.31	0.580
No	166 (97.65)	80 (96.39)	86 (98.85)		
Yes	4 (2.35)	3 (3.61)	1 (1.15)		
Variables	Total cohort (n=170)	Non-MM group (n=83)	MM group (n=87)	Statistic	P
ROS1, n (%)				-	0.237
No	168 (98.82)	81 (97.59)	87 (100.00)		
Yes	2 (1.18)	2 (2.41)	0 (0.00)		
ERBB, n (%)				-	0.488
No	169 (99.41)	82 (98.80)	87 (100.00)		
Yes	1 (0.59)	1 (1.20)	0 (0.00)		
EGFR amplification, n (%)				χ²=2.59	0.108
No	163 (95.88)	77 (92.77)	86 (98.85)		
Yes	7 (4.12)	6 (7.23)	1 (1.15)		
RET, n (%)				-	0.237
No	168 (98.82)	81 (97.59)	87 (100.00)		
Yes	2 (1.18)	2 (2.41)	0 (0.00)		
MET, n (%)				χ²=0.31	0.580
No	166 (97.65)	80 (96.39)	86 (98.85)		
Yes	4 (2.35)	3 (3.61)	1 (1.15)		
MCHC, M (Q₁, Q₃)	333.00 (326.18, 337.00)	334.00 (327.95, 339.70)	330.00 (324.00, 336.20)	Z=-2.09	0.036
PTA, M (Q₁, Q₃)	105.00 (97.90, 116.30)	107.00 (100.55, 120.40)	104.00 (95.00, 114.00)	Z=-2.00	0.046
TT, M (Q₁, Q₃)	15.00 (13.70, 16.38)	15.50 (14.30, 16.60)	14.50 (13.50, 16.10)	Z=-2.34	0.019
ALP, M (Q₁, Q₃)	96.00 (76.00, 117.00)	90.00 (71.00, 109.50)	100.00 (78.00, 123.50)	Z=-2.01	0.044
DBIL, M (Q₁, Q₃)	3.44 (2.63, 4.30)	3.30 (2.60, 3.88)	3.60 (2.95, 4.60)	Z=-2.24	0.025

BBB: blood-brain barrier; TKIs: tyrosine kinase inhibitors; EGFR: epidermal growth factor receptor; TP53: tumor protein p53; BRAF: v-raf murine sarcoma viral oncogene homolog B; RB1: retinoblastoma protein 1; ROS1: c-ros oncogene 1 receptor kinase; ERBB: erythroblastic leukemia virus B; RET: rearranged during transfection; MET: mesenchymal-epithelial transition factor; MCHC: mean corpuscula rhemoglobin concentration; ALP: alkaline phosphatase; DBIL: direct bilirubin.

### 2.2 LASSO回归因子筛选

对全部88个变量进行了LASSO回归分析（图2B、2C）。在LASSO回归模型中，我们采用交叉验证方法，并通过“cv.glmnet”函数确定了使交叉验证（α=1, k=10）误差最小的最优λ值（λ=0.03061）。结果显示，年龄、位置分布、合并肾上腺结节或增生、肝转移、下肢骨转移、胸廓骨转移、癌性淋巴管炎、阻塞性肺炎、EGFR L858R、ALK、TP53、ROS1基因突变、EGFR拷贝数扩增、MM前应用高血脑屏障透过率的TKIs治疗、抗血管治疗、免疫治疗、基线时MCHC、凝血酶原活动度（prothrombin time activity, PTA）、TT、GLB、DBIL水平这21项重要变量可作为非手术干预的LUAD患者发生MM的预测因子。

### 2.3 单因素及多因素Logistic回归分析结果

将LASSO回归筛选出来的变量纳入单因素Logistic回归分析（表4），得出确诊肺癌时的年龄（P=0.033）、位置分布（P=0.010）、肝转移（P=0.003）、下肢骨转移（P=0.006）、癌性淋巴管炎（P=0.017）、阻塞性肺炎（P=0.010）、EGFR L858R突变（P=0.028）、ALK突变（P=0.015）、TP53突变（P=0.045）、基线时PTA水平（P=0.045）、TT水平（P=0.015）、DBIL水平（P=0.023）、GLB水平（P=0.039）以及MM前是否应用免疫检查点抑制剂治疗（P=0.034）是影响MM发生的相关因素。将上述P<0.05的变量进一步纳入多因素Logistic回归模型分析（表4）。结果提示: 年龄（OR=0.93, 95%CI: 0.89-0.98, P=0.004）、EGFR L858R突变（OR=2.94, 95%CI: 1.21-7.48, P=0.008）、ALK突变（OR=0.09, 95%CI: 0.01-0.63, P=0.040）、基线时合并肝转移（OR=17.34. 95%CI: 3.01-162.64, P=0.004）、基线GLB水平（OR=0.88, 95%CI: 0.80-0.97, P=0.039）是罹患MM的独立风险因素。存在EGFR L858R突变、初诊时合并肝转移的患者罹患MM的风险增加，基线时存在ALK基因突变以及较高的GLB水平的患者罹患MM的风险下降。此外，低龄的患者罹患MM的风险增加。

**表4:** 170例LUAD患者的单因素及多因素Logistic回归结果

Variables	Univariate Logistic regression						Multivariate Logistic regression				
	β	S.E	Z	P	OR (95%CI)		β	S.E	Z	P	OR (95%CI)
Age	-0.04	0.02	-2.14	0.033	0.96 (0.93-0.99)		-0.07	0.02	-2.90	0.004	0.93 (0.89-0.98)
Tumor location											
Central type					1.00 (Reference)						1.00 (Reference)
Peripheral type	-0.81	0.31	-2.58	0.010	0.45 (0.24-0.82)		-0.73	0.42	-1.74	0.082	0.48 (0.21-1.09)
Cancerous lymphangitis											
No					1.00 (Reference)						1.00 (Reference)
Yes	1.87	0.78	2.40	0.017	6.48 (1.70-42.53)		1.46	0.96	1.53	0.127	4.31 (0.74-36.08)
Obstructive pneumonia											
No					1.00 (Reference)						1.00 (Reference)
Yes	0.95	0.37	2.58	0.010	2.59 (1.28-5.48)		0.67	0.53	1.25	0.211	1.95 (0.69-5.66)
Lower limb bone metastasis											
No					1.00 (Reference)						1.00 (Reference)
Yes	1.14	0.41	2.76	0.006	3.11 (1.43-7.24)		0.44	0.56	0.80	0.426	1.56 (0.53-4.84)
Liver metastases											
No					1.00 (Reference)						1.00 (Reference)
Yes	2.29	0.77	2.99	0.003	9.84 (2.69-63.41)		2.85	0.99	2.87	0.004	17.34 (3.01-162.64)
EGFR L858R mutations											
No					1.00 (Reference)						1.00 (Reference)
Yes	0.73	0.33	2.20	0.028	2.08 (1.09-4.05)		1.08	0.46	2.33	0.008	2.94 (1.21-7.48)
TP53 mutations											
No					1.00 (Reference)						
Yes	0.84	0.42	2.01	0.045	2.32 (1.04-5.49)		0.57	0.54	1.06	0.287	177 (0.63-5.30)
ALK mutations											
No					1.00 (Reference)						1.00 (Reference)
Yes	-2.58	1.06	-2.44	0.015	0.08 (0.01-0.40)		-2.41	1.17	-2.06	0.040	0.09 (0.01-0.63)
Immunotherapy											
No					1.00 (Reference)						1.00 (Reference)
Yes	-0.78	0.37	-2.12	0.034	0.46 (0.22-0.93)		-0.68	0.50	0.53	0.200	1.95 (0.69-5.66)
PTA (70%-150%)	-0.02	0.01	-2.01	0.045	0.98 (0.96-0.99)		-0.02	0.01	-1.85	0.064	0.98 (0.95-1.00)
TT (10-18 s)	-0.22	0.09	-2.43	0.015	0.81 (0.67-0.96)		-0.21	0.12	-1.79	0.073	0.81 (0.63-1.00)
GLB (20-35 g/L)	-0.07	0.03	-2.07	0.039	0.93 (0.87-0.99)		-0.13	0.49	-2.57	0.039	0.88 (0.80-0.97)
DBIL (0-10 μmol/L)	0.27	0.12	2.27	0.023	1.31 (1.04-1.67)		0.21	0.16	1.33	0.184	1.23 (0.92-1.70)

OR: odds ratio; CI: confidence interval; GLB: globulin.

在多因素Logistic回归模型中，我们通过特征重要性图对每个输入特征对模型预测结果的贡献程度进行了可视化分析（图3）。图中特征的长度代表其对模型预测结果的贡献程度，长度越长表示贡献越大。特征的方向（正向或负向）代表其与目标变量之间的关系方向。可以看出，对模型贡献程度最高的三个变量为肝转移、ALK以及EGFR L858R突变，对模型的贡献度分别为3.12、-2.33以及1.05。

**图3:**
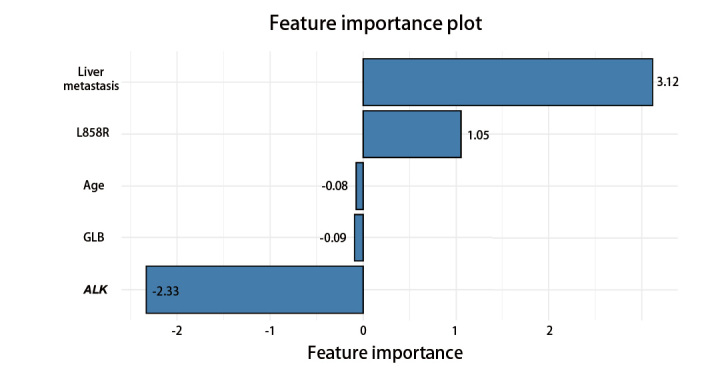
特征重要性图。图中显示了各个特征的重要性，每个特征旁边标注了其重要性得分。

### 2.4 多因素Logistic回归模型的评价

随后，我们分别绘制年龄、EGFR L858R突变状态、ALK突变状态、肝转移状态与GLB的受试者工作特征（receiver operating characteristic, ROC）曲线，结果显示年龄的曲线下面积（area under the curve, AUC）为0.583（95%CI: 0.497-0.668）、EGFR L858R突变的AUC为0.580（95%CI：0.510-0.651）、ALK突变的AUC为0.561（95%CI：0.522-0.599）、肝转移的AUC为0.586（95%CI：0.541-0.631）与基线GLB的AUC为0.586（95%CI：0.500-0.671）。利用上述变量构建联合模型，计算出AUC为0.855（95%CI: 0.800-0.909），表明模型具有较高的区分度，且联合预测指标的检验效能要明显优于单独预测指标（图4A）。校准曲线与参考对角线的偏差处于可接受范围内，平均绝对误差为0.056（图4B）。Hosmer-Lemeshow检验计算出P值为0.445，表明模型的预测概率能够准确反映实际概率。此外，临床决策曲线及临床影响图还表明基于联合模型的干预决策具有明显的临床益处（图5）。

**图4:**
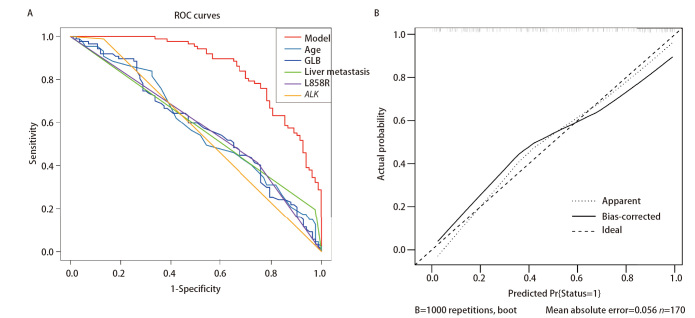
模型的区分度和准确度的评价。A：单变量及多变量模型的ROC曲线；B：多因素Logistic回归模型的校准曲线

**图5:**
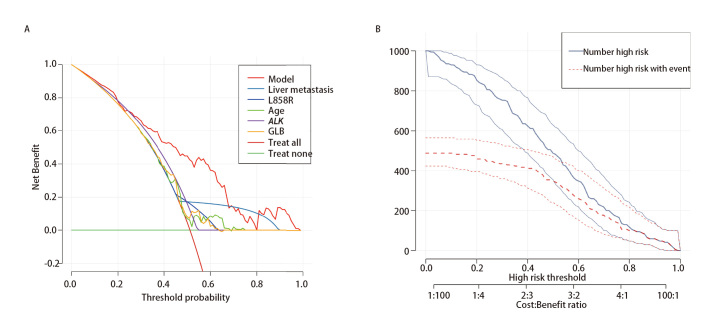
临床评价图。A：单个变量及综合模型临床决策曲线；B：基于多因素Logistic回归模型临床影响图。

## 3 讨论

MM的预后极差，因此对具有高MM风险患者的早期识别至关重要。目前关于MM预测模型的研究相对有限。Li等^[5]^的研究纳入了102例非小细胞肺癌（non-small cell lung cancer, NSCLC）MM患者，发现EGFR 21外显子L858R突变在MM患者中的比例显著较高。该研究构建的预测模型的C指数为0.767，表明其具有一定的预测能力，但相较于本研究中构建的模型，其性能略逊一筹。梁平安^[6]^针对一线接受TKIs药物治疗的NSCLC MM患者构建了预测模型，其C指数为0.833，与本研究中的模型性能相近，表明两者在预测MM方面具有相似的效用。本研究回顾性分析了170例非手术干预的LUAD患者的临床资料。通过LASSO回归、单因素及多因素Logistic回归分析，我们发现基线时存在肝转移和L858R突变是LUAD患者发生MM的独立危险因素，这一结果与梁^[6]^的研究一致。肝脏作为高度免疫耐受的器官，其解剖结构具有压力小、血流速度较慢等血流动力学特点，使得肿瘤细胞易于在肝脏内滞留并定植^[7]^。然而，肺癌肝转移的发生率并未如预期般常见^[8]^。这可能与肝脏的微环境有关。肝脏微环境中的Kupffer细胞（Kupffer cell, KC）和肝窦内皮细胞（liver sinusoidal endothelial cell, LSEC）在肿瘤肝转移过程中发挥着双重作用^[9]^。KC是肝脏内的长期驻留型巨噬细胞，LSEC则是最早接触肿瘤细胞的肝脏固有细胞^[10]^。当肿瘤负荷较低时，KC和LSEC能够通过释放细胞因子和趋化因子激活自然杀伤细胞和中性粒细胞，或分泌肿瘤坏死因子、一氧化氮和活性氧等物质，通过Fas-FasL途径促进肿瘤细胞的死亡，共同形成了抵御肿瘤细胞入侵肝脏的屏障。然而，随着肿瘤负荷的增加，这些细胞逐渐转变为促进肿瘤细胞在肝脏定植的角色。具体而言，KC可分泌血管内皮生长因子（vascular endothelial growth factor, VEGF）和基质金属蛋白酶（matrix metalloproteinases, MMP），其中高水平的VEGF-A导致周细胞脱落和脑微血管内皮细胞增殖，能够损伤血脑屏障（blood-brain barrier, BBB）的完整性^[11]^。值得注意的是，在MM患者的脑脊液中，也检测到显著升高的VEGF浓度，这进一步支持其在BBB损伤和疾病进展中的关键作用^[12]^。此外，肿瘤细胞产生的MMP通过降解致密基质促进肿瘤细胞迁移，被认为是破坏BBB的主要因素之一^[13-16]^。LSEC还能促使结直肠癌细胞表达细胞间质上皮转换因子（cellular-mesenchymal epithelial transition factor, c-Met），增强其侵袭能力，进一步促进肿瘤细胞的增殖、血管生成及加速其对肝实质的入侵^[17]^。因此，基线时合并肝转移可能预示着较大的肿瘤负荷、更强的侵袭能力和BBB的破坏。值得注意的是，本研究中肝转移的95%CI较宽，可能受到样本量不足的影响，需要进一步增加样本量来验证这一结果。

尽管19Del是LUAD最常见的基因突变，但在MM中，L858R突变则更为常见^[18]^。我们推测这一现象可能与L858R与19Del对PI3K-AKT-mTOR信号通路中的影响不同有关。这是由于L858R突变常伴随其他基因突变，并能够激活CXCL12-CXCR4通路促进MMP的表达，增强LUAD细胞的侵袭能力^[19,20]^。此外，二者对EGFR-TKIs治疗的反应也有较大的差别。具体而言，19Del突变导致EGFR蛋白N端结构域缩短并促进其与αC螺旋的结合，而L858R突变则发生在C端结构域，影响了其与αC螺旋的相互作用^[21]^。这种空间构型的差异直接导致了二者对EGFR-TKIs亲和力的不同。相较于19Del，L858R突变对EGFR-TKI的亲和力更低，选择性更差，因此其对EGFR-TKIs治疗的反应也较差^[22]^。目前关于L858R突变在MM中的作用机制研究较为有限，未来对该领域的研究或许能够改善EGFR L858R突变MM患者的预后。

既往研究^[23,24]^表明，ALK基因突变与神经系统转移密切相关。在一项针对291例ALK重排肺癌患者的研究中发现，MM的发生率高达10.3%。一方面，ALK突变能够介导包括PLCγ、JAK-STAT、PI3K-AKT、mTOR及MAPK等信号通路，促进肿瘤细胞的生长、侵袭与转移^[25]^。另一方面，ALK抑制剂阿来替尼（Alectinib）和洛拉替尼（Lorlatinib）能够有效抑制与上皮间质转化（epithelial-mesenchymal transition, EMT）相关的蛋白以及MMP的mRNA和蛋白质的表达，降低活性氧水平、减少脂质过氧化以及氧化DNA损伤，并且灭活血管中的核苷酸结合寡聚结构域样受体蛋白3（nucleotide-binding oligomerization domain-like receptor family pyrin domain containing 3, NLRP3）炎性小体通路，从而减轻脑血管完整性丧失并保护BBB免受损伤^[26,27]^。这一结果与本研究中基线时存在ALK基因突变的患者MM风险较低的发现不一致。这种差异可能归因于近年来针对ALK的靶向药物能够有效穿越BBB，从而降低了MM的发生率。在本研究中，MM组中有1例患者检测到ALK基因突变阳性。该患者在接受MM诊断前曾接受一代ALK抑制剂克唑替尼（Crizotinib）的口服治疗。而在未发生MM的11例ALK突变患者中，2例患者接受了洛拉替尼的口服治疗，1例患者接受了赛瑞替尼（Ceritinib）的口服治疗，其余8例患者则接受了阿来替尼的口服治疗。然而，受到样本数量的限制，未来需要更大规模的前瞻性研究以进一步验证和拓展本研究的发现。

血清GLB能够通过经典补体途径激活补体系统，并调节抗体依赖性细胞介导的细胞毒性，从而有效清除肿瘤细胞，在一定程度上反映机体的免疫状态。既往研究^[24,28]^显示年龄对肿瘤细胞的生物学行为有显著影响。在本研究中，年龄被确定为MM的一个保护性因素，这可能与年龄对肿瘤细胞生物学特性的影响有关。随着年龄的增长，由于免疫器官出现结构的破坏和重塑以及随后的先天性和适应性免疫功能障碍，老年患者更容易患癌^[29]^。然而，在多种类型的老年肿瘤患者中，尽管存在免疫衰老现象，但由于肿瘤细胞也受到宿主衰老因素的影响，并未表现出更具侵袭性的表型。相比之下，年轻患者中的肿瘤细胞则更容易发生转移和扩散，展现出更强的侵袭性生物学特征^[24,28]^。

然而，本研究存在若干局限性需予以关注：首先，本研究仅纳入了脑脊液细胞学阳性或通过头颅增强MRI确诊为MM的患者。这一选择标准可能使研究结果不完全适用于仅有神经系统症状但脑脊液和影像学检查结果均为阴性的MM患者。其次，MM包括软MM和硬MM两种亚型，而本研究未能对这两种亚型进行区分，这可能影响研究结果的普遍适用性。因此，未来的研究应着重探讨不同类型的MM。最后，本研究为单中心回顾性研究，样本量有限，缺乏外部数据验证，因此存在一定的选择偏倚。鉴于此，未来可以通过扩大样本量，采用多中心合作以及前瞻性研究设计来进一步验证本研究的结论。

综上，本研究显示，低龄、携带EGFR 21外显子L858R突变以及初诊时合并肝转移是非手术干预的LUAD患者罹患MM的独立危险因素，而基线时存在ALK基因突变以及较高的GLB水平的高龄患者较不容易罹患MM。因此，在临床上对于确诊LUAD时年龄较小、携带L858R基因突变、基线时合并肝转移的非手术干预的LUAD患者应尽早实施MM的三级预防措施，并定期监测患者的MM状态。
